# Untargeted Multiomics Approach Coupling Lipidomics and Metabolomics Profiling Reveals New Insights in Diabetic Retinopathy

**DOI:** 10.3390/ijms241512053

**Published:** 2023-07-27

**Authors:** Patricia Ancel, Jean Charles Martin, Elisa Doukbi, Marie Houssays, Pierre Gascon, Maud Righini, Frédéric Matonti, Ljubica Svilar, Marie Valmori, Catherine Tardivel, Nicolas Venteclef, Jean Baptiste Julla, Jean François Gautier, Noémie Resseguier, Anne Dutour, Bénédicte Gaborit

**Affiliations:** 1 Aix-Marseille University, INSERM, INRAE, C2VN, 13005 Marseille, France; patricia.ancel@univ-amu.fr (P.A.); elisa.doukbi@univ-amu.fr (E.D.); 2 Aix-Marseille University, INSERM, INRAE, C2VN, BIOMET Aix-Marseille Technology Platform, 13005 Marseille, France; jean-charles.martin@univ-amu.fr (J.C.M.); marie.valmori@univ-amu.fr (M.V.); catherine.tardivel@univ-amu.fr (C.T.); 3 Medical Evaluation Department, Assistance-Publique Hôpitaux de Marseille, CIC-CPCET, 13005 Marseille, France; 4 Department of Ophthalmology, Assistance-Publique Hôpitaux de Marseille, 13005 Marseille, France; pierre.gascon3@gmail.com (P.G.); mrighini@hopital-saint-joseph.fr (M.R.); frederic.matonti@free.fr (F.M.); 5 Centre Monticelli Paradis, 433 bis rue Paradis, 13008 Marseille, France; 6 Groupe Almaviva Santé, Clinique Juge, 116 rue Jean Mermoz, 13008 Marseille, France; 7 CRIBIOM Aix-Marseille Technology Platform, 13005 Marseille, France; ljubica.svilar@univ-amu.fr; 8 IMMEDIAB Laboratory, Institut Necker Enfants Malades (INEM), INSERM U1151, CNRS UMR 8253, Université Paris Cité, 75015 Paris, France; nicolas.venteclef@inserm.fr; 9 IMMEDIAB Laboratory, Diabetology and Endocrinology Department, Institut Necker Enfants Malades (INEM), INSERM U1151, CNRS UMR 8253, Université Paris Cité, Lariboisière Hospital, Féderation de Diabétologie, APHP, 75015 Paris, France; jeanbaptiste.julla@aphp.fr (J.B.J.); jean-francois.gautier@aphp.fr (J.F.G.); 10 Aix-Marseille University, Support Unit for Clinical Research and Economic Evaluation, Assistance Publique-Hôpitaux de Marseille, EA 3279 CEReSS-Health Service Research and Quality of Life Center, 13005 Marseille, France; noemie.resseguier@univ-amu.fr; 11 Aix-Marseille University, INSERM, INRAE, C2VN, Endocrinology, Metabolic Diseases and Nutrition Department, AP-HM, 13005 Marseille, France; anne.dutour@ap-hm.fr

**Keywords:** diabetic retinopathy, metabolomics, lipidomics

## Abstract

Diabetic retinopathy (DR) is a microvascular complication of diabetes mellitus (DM) which is the main cause of vision loss in the working-age population. Currently known risk factors such as age, disease duration, and hemoglobin A1c lack sufficient efficiency to distinguish patients with early stages of DR. A total of 194 plasma samples were collected from patients with type 2 DM and DR (moderate to proliferative (PDR) or control (no or mild DR) matched for age, gender, diabetes duration, HbA1c, and hypertension. Untargeted lipidomic and metabolomic approaches were performed. Partial-least square methods were used to analyze the datasets. Levels of 69 metabolites and 85 lipid species were found to be significantly different in the plasma of DR patients versus controls. Metabolite set enrichment analysis indicated that pathways such as metabolism of branched-chain amino acids (methylglutaryl carnitine *p* = 0.004), the kynurenine pathway (tryptophan *p* < 0.001), and microbiota metabolism (p-Cresol sulfate *p* = 0.004) were among the most enriched deregulated pathways in the DR group. Moreover, Glucose-6-phosphate (*p* = 0.001) and N-methyl-glutamate (*p* < 0.001) were upregulated in DR. Subgroup analyses identified a specific signature associated with PDR, macular oedema, and DR associated with chronic kidney disease. Phosphatidylcholines (PCs) were dysregulated, with an increase of alkyl-PCs (PC O-42:5 *p* < 0.001) in DR, while non-ether PCs (PC 14:0–16:1, *p* < 0.001; PC 18:2–14:0, *p* < 0.001) were decreased in the DR group. Through an unbiased multiomics approach, we identified metabolites and lipid species that interestingly discriminate patients with or without DR. These features could be a research basis to identify new potential plasma biomarkers to promote 3P medicine.

## 1. Introduction

Type 2 Diabetes mellitus (T2D) is a chronic disease that is increasing worldwide with the diabesity pandemic, leading to poor health outcomes and high health care costs. Diabetic retinopathy (DR) remains a common complication of DM which affects 22.3% of people living with diabetes [1,2]. It is estimated that in 2030, the number of adults worldwide with DR will be 129.84 million, and this number is projected to increase to 160.50 million in 2045 [1]. Despite prevention, DR remains a leading cause of blindness in the adult working population [1]. Currently known risk factors such as age, disease duration, and hemoglobin A1c lack the efficiency to distinguish patients with early stages of DR [2,3]. Furthermore, HbA1c accounts for only 6.6% of the variation in DR risk [4]. A recent systematic review pointed to 6.6% (interquartile range 1.9–9.8%) DR prevalence in patients with prediabetes, suggesting that DR diagnosis is often preceded by a long and false silent phase [5]. Frequent retinal screening for all people living with diabetes is an effective method of preventing DR complications. However, many patients have ocular comorbidities such as cataracts, which impedes clinical diagnosis [6]. This differentiation relies on of ophthalmologists; however, due to shortages of human resources for eye health, there is an important challenge in the coming years to better predict patients at risk for developing vision-threatening stages of DR [7]. The Angiosafe T2D cohort is a French national prospective cohort aiming to include 7200 patients who will be followed up at three years to evaluate DR presence, incidence, and progression as well as new biomarkers (angiogenic, epigenomic, and proinflammatory) of DR progression [8]. Lipidomics and metabolomics, or the comprehensive profiling of lipid species and metabolites, respectively, in biological systems, has undergone a rapid technological evolution within the past decade. These advances have led to the application of multiomics approaches to defining trajectories associated with T2D by integrating the impact of silent microvascular damage through cohorts of patients using high-throughput longitudinal phenotyping [9]. Metabolomics is a novel high-throughput profiling technique that can comprehensively reveal the levels of small-molecule metabolites of size <1500 Da [10]. Because the metabolome is downstream from the genome, transcriptome, and proteome, it represents a more sensitive level of organization for understanding complex biological systems, and can provide a highly integrated profile of biological status [9,11]. Lipidomics focuses on non-polar metabolites on a large scale based on analytical chemistry principles and technological tools, particularly mass spectrometry (MS) [12]. Recently, an increasing number of studies have used both metabolomics and lipidomics to investigate potential biomarkers and mechanisms, expanding understanding of the pathogenesis of microvascular complications of diabetes [13].

In the current study, plasma samples were collected from 200 T2DM patients from the Angiosafe T2D cohort aged 42–80 years, including 100 patients with moderate to proliferative (PDR) and 100 with mild or no DR. Untargeted metabolomics and lipidomics were comprehensively analyzed using these plasma samples. Bioinformatics analysis was then performed to decipher possible relevant pathways.

## 2. Results

### 2.1. Study Population

In total, 194 patients were analyzed (97 control and 97 DR) after exclusion of six patients due to poor analytical data. Table 1 shows the main characteristics of patients matched by age (±5 years), sex, duration of diabetes (±2 years), HbA1c (±0.5%), and hypertension. As expected, patients in the DR group were more likely to have nephropathy than patients in the control group. There was no significant difference in the number of patients treated with GLP-1 analogs in the two groups (*p* = 0.99).

### 2.2. Feature Detection in Metabolomics and Lipidomics Analyses

**Figure 1:**
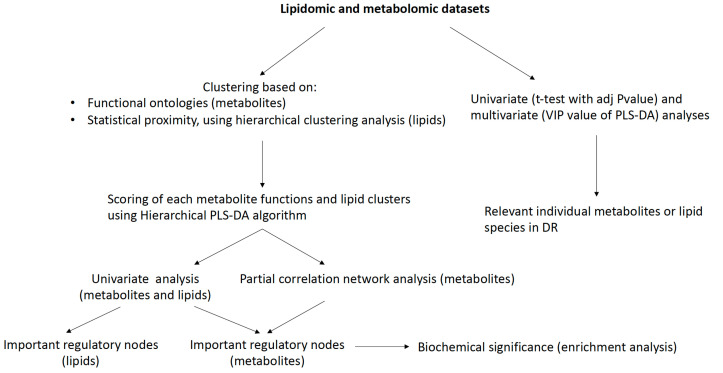
Statistical workflow.

After removing blank peaks, performing normalization and coefficient of variation filtering, and removing overlapping identification from both ionization modes, a total of 228 metabolites were annotated from our in-house libraries. The lipid datasets contained 335 annotated lipid species. Thus, a total of 563 annotated variables were obtained and retained for each patient and used for further statistical analyses (Figure 1). A summary of the identified metabolites detected with assigned metabolic functional groupings for multiblock analysis and annotated lipid species are provided in the Supplementary Data.

### 2.3. Multiblock Analysis

The 228 annotated metabolites were clustered into 54 functional biological blocks, as described in the methods section. Lipids were blocked according to their statistical proximity using hierarchical clustering analysis (335 lipid species clustered into eleven different blocks; see Supplementary Data). Grouping metabolites or lipid species sharing the same biological functions or statistical proximity allows the complexity of the dataset to be simplified and facilitates data interpretation.

#### 2.3.1. Multiomics Analysis

**Figure 2:**
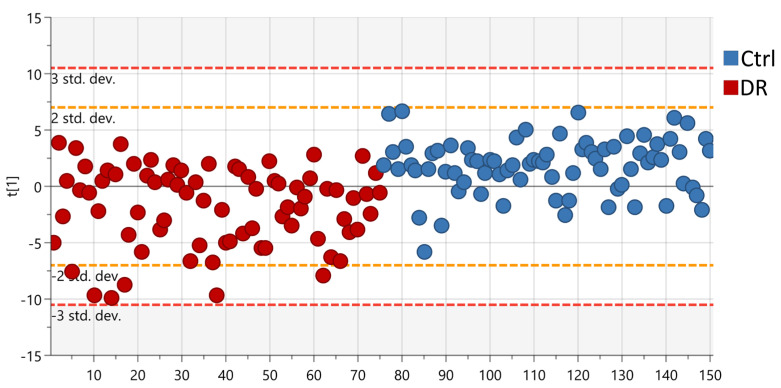
Partial least-squares discriminant analysis (PLS−DA) score plot based on multiomics clustering analysis. The red spots represent samples from the DR group and the blue spots represent samples from the Control group.

**Figure 3:**
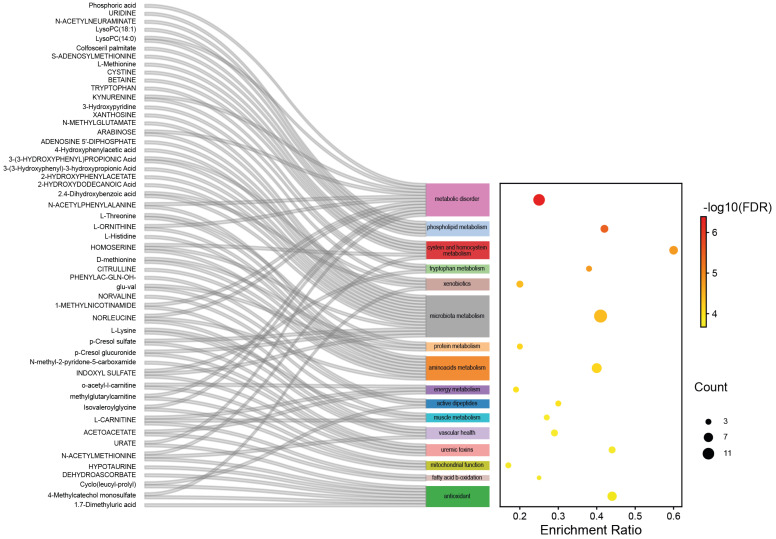
Top sixteen metabolic functions differentially regulated between the DR and Control groups.

A set of 26 biological functions and seven lipid clusters were found to be differentially regulated between the DR and Control groups using a statistical multi-test procedure (Variable of Importance in Projection of a partial-least square discriminant analysis (PLS-DA) followed by a *t*-test. The R2 and Q2 of the final PLS-DA model comprising all the individuals and based on these 33 clusters were 0.39 and 0.25 (*p* < 9 × 10^−10^), respectively (Figure 2). In particular, phospholipids, tryptophan, amino acids, and energy metabolism were found to be the main metabolic functions dysregulated in DR patients compared to control group patients (FDR < 0.0001), along with vascular health, mitochondrial function, uremic toxins, and antioxidant function (Figure 3).

#### 2.3.2. Metabolic Functions

**Figure 4:**
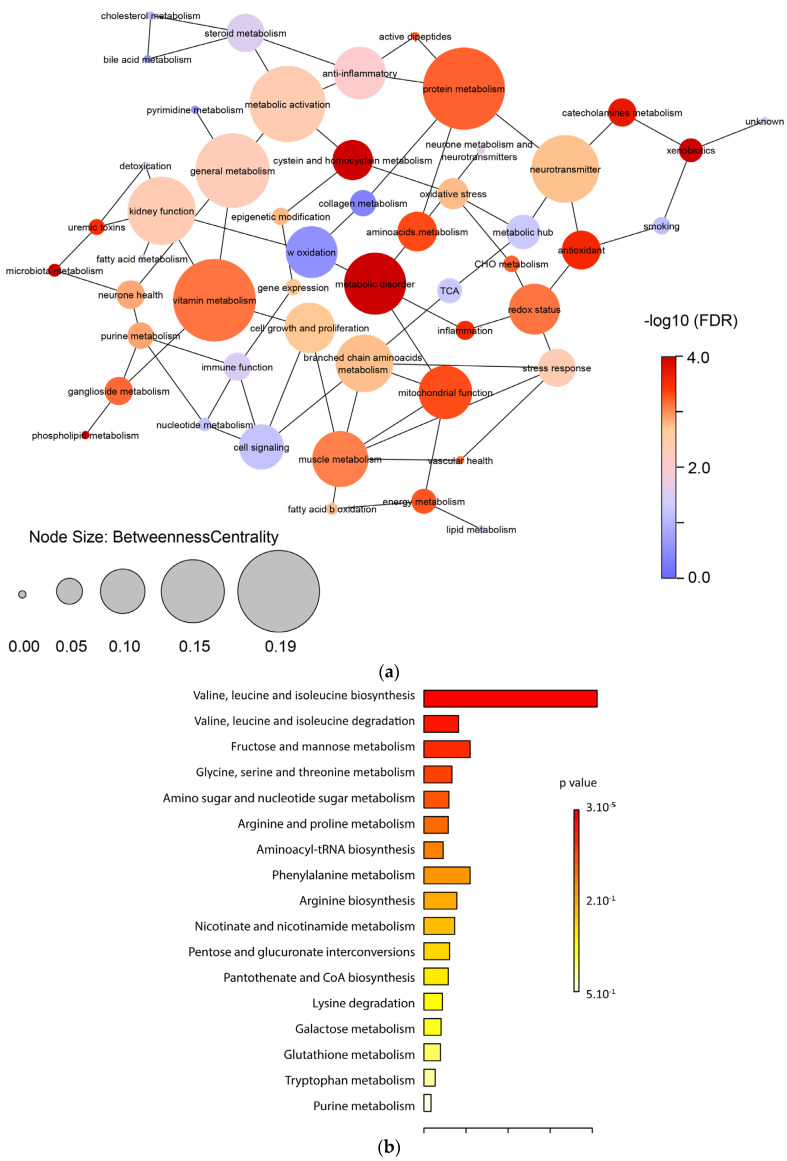
(**a**) Partial correlation network integrating all the metabolic functions, displaying interactions and betweenness centrality. The node color corresponds to the statistical significance between the DR and Control groups. The node size relates to the value of the betweenness centrality coefficients calculated in Cytoscape. Initial partial correlations were defined as *p* ≤ 0.25. A node with higher betweenness centrality has more weight in the network. Thus, in addition to the statistical significance, this element of network topology permits an appreciation of the importance of specific metabolic regulations in biological systems. (**b**) KEGG enrichment analysis of the central node “metabolic disorder”.

Because metabolic regulations rarely occur independently [14], we calculated a partial correlation network integrating all the biological functions (Figure 4). Correlation network analysis of these data revealed that protein (FDR = 7.3 × 10^−5^), vitamin metabolism (FDR = 2.5 × 10^−5^), and metabolic disorder (FDR = 4.1 × 10^−7^) had a higher degree of connection or betweenness centrality score than others. To decipher the extent to which molecular regulations of the metabolites of the metabolic disorder function correspond, we performed a KEGG enrichment analysis on the metabolites forming this function. It showed that branched-chain amino acid (valine, leucine, isoleucine) biosynthesis and degradation (*p* = 3 × 10^−5^ and *p* = 0.005, respectively) and fructose and mannose metabolism (*p* = 0.013) ranked highest in terms of frequency in the DR group (Figure 4).

#### 2.3.3. Lipid Clusters

**Figure 5:**
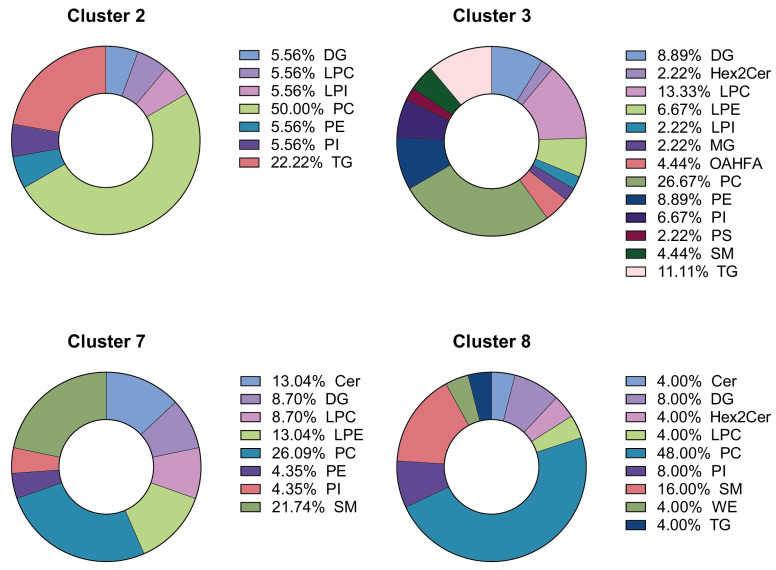
Lipid composition of the top four lipid clusters differentially regulated between the DR and Control groups. Cer: ceramides, DG: diglycerides, Hex2Cer: dihexosylceramide, LPC: lisophophatidylcholine, LPE: lisophophatidylethanolamine, LPI: lisophophatidylinositol, MG: monoglycerides, OAHFA: (O-Acyl)-ω-hydroxy fatty acids, PC: phosphatidylcholine, PE: phophatidylethanolamine, PI: phophatidylinositol, PS: phophatidylserine, SM: sphingomyelin, TG: triglycerides, WE: wax ester.

Seven out of eleven lipid clusters were significantly highlighted in the DR group. The top four (Clusters 2, 3, 7, and 8) are represented in Figure 5. They include triglycerides (TG), diglycerides (DG), phosphatidylcholine (PC), phosphatidylethanolamine (PE), sphingomyelin (SM), ceramide (Cer), and phosphatidylinositol (PI). Cluster 2 is composed mainly of PC (50%) and TG. Cluster 3 is composed mainly of PC (27% and LPC (13%). Clusters 7 and 8 are composed mainly of PC (26% and 48%, respectively) and SM (22% and 16%, respectively).

### 2.4. Specific Metabolites and Lipids

**Figure 6:**
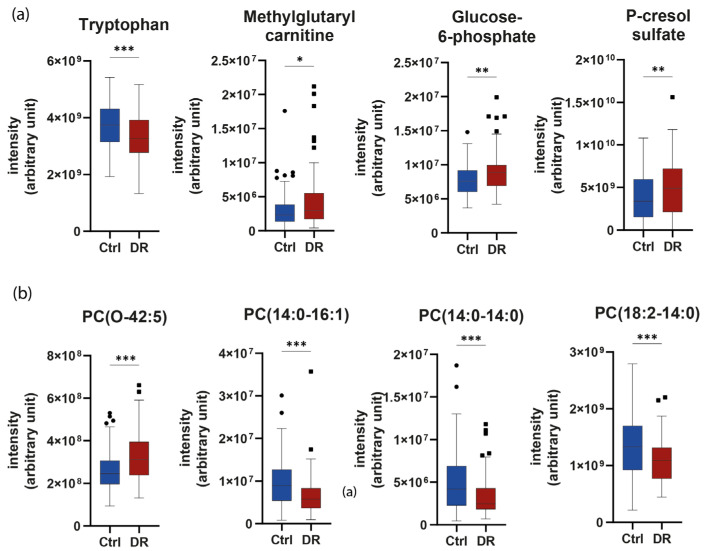
Top four metabolites (**a**) and lipid species (**b**) between the DR and Control groups. PC: phosphatidylcholine. * *p* < 0.05, ** *p* < 0.01, *** *p* < 0.001.

We then focused on the most specific individual polar and apolar metabolites as potent biomarkers of DR. For this, we used the metabolites selected by both the variable of importance in projection (VIP) of the PLS algorithm and a *t*-test to determine the Top 4 significant metabolites and lipid species in our previous multiomics signature of DR (Figure 6). Tryptophan (fold change −1.12, VIP = 2.59, *p* < 0.001) was significantly reduced in the plasma of the DR group, whereas methylglutarylcarnitine (fold change 1.59, VIP = 2.13, *p* = 0.004), Glucose-6-Phosphate (fold change 1.13, VIP = 2.07, *p* = 0.001), and the uremic toxin p-Cresol sulfate (fold change 1.47, VIP = 1.98, *p* = 0.008) were significantly upregulated in the plasma of the DR group in comparison to the control group.

Regarding lipid species, PCs were the most dysregulated in DR. PC(O-42:5) (fold change 1.23, VIP = 2.60, *p* < 0.001) was found to be significantly increased in DR vs. the control group. By contrast, three PCs containing a myristic acid (14:0), i.e., PC(14:0–16:1) (fold change −1.43, VIP = 2.49, *p* < 0.001), PC(14:0–14:0) (fold change −1.47, VIP = 2.44, *p* < 0.001), and PC(18:2–14:0) (fold change −1.23, VIP = 2.52, *p* < 0.001) were found to be significantly decreased in the DR group compared to the control group. Lastly, ether PCs were upregulated (*p* = 0.009), while non-ether PCs were found to be lower (*p* = 0.022) in the DR group compared to control. Regarding sphingolipids, Cer(d18:2_24:1) (fold change = 1.20 VIP = 2.16 *p* = 0.007), SM(d33:1) (fold change = 1.06 VIP = 1.46 *p* = 0.026), SM(d34:2) (fold change = 1.09 VIP = 1.80 *p* = 0.014), and SM(d36:5) (fold change = 1.11 VIP = 2.06 *p* = 0.005) were found to be significantly elevated in the DR group compared to the control group.

### 2.5. Impact of Diabetic Kidney Disease

To determine whether nephropathy could affect the association with DR, subgroup analyses of the control (*n* = 61 patients without DR and without DKD), DR only (i.e., DR without DKD) (*n* = 44), diabetic kidney disease (DKD) only (i.e., DKD without DR) (*n* = 36), and DR + DKD (*n* = 53) subgroups were performed using metabolomic data. Twenty-five common metabolites, including N-methylglutamate, glucose-6-phosphate, tryptophan, and kynurenic acid, were found both in the DR and the nephropathy group. Interestingly, in the DR-only subgroup (i.e., DR without nephropathy), methylglutarylcarnitine (fold change 1.86, VIP = 2.12, *p* = 0.037) was found to be significantly increased compared to control, as previously found in the multiomics signature (Table 2).

In addition, we identified a specific metabolomic signature for the DR + DKD subgroup involving a significant increase in methylguanidine (*p* < 0.001, fold change 1.89), N-acetylneuraminate (*p* < 0.001, fold change 1.73), arabinose (*p* < 0.001, fold change 1.70), and mevalolactone (*p* < 0.001, fold change 1.30) compared to the control group (Table 2).

### 2.6. Specific Signatures of Diabetic Eye Disease

**Figure 7:**
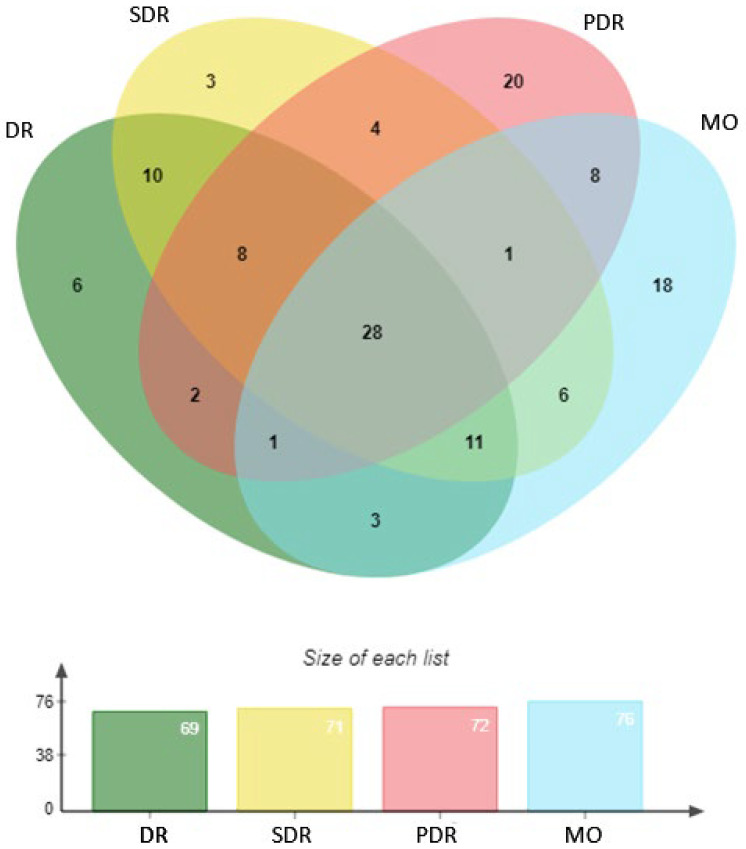
Venn diagram representing each metabolic signature of diabetic eye disease in different subgroup populations vs. patients with no diabetic retinopathy (DR). DR included mild, moderate, severe, proliferative, and macular oedema, while SDR (severe diabetic retinopathy) comprised moderate to proliferative DR diagnosis and macular oedema (MO), PDR (proliferative diabetic retinopathy), or MO (macular oedema).

As patients with diabetes may have different stages of DR and these may or may not be associated with macular oedema, we performed a new subgroup analysis with the metabolomics data to determine the specificity of each eye disease compared to patients with no DR (*n* = 80). Four eye diseases were determined: diabetic retinopathy (DR), including mild, moderate, severe, proliferative, and macular oedema (MO) (*n* = 114); and severe diabetic retinopathy (SDR) comprising either moderate to proliferative DR diagnosis and macular oedema (*n* = 97), proliferative diabetic retinopathy (PDR) (*n* = 24), or macular oedema (MO) (*n* = 30). In all, 69, 71, 72, and 76 metabolites with a VIP > 1.0 were respectively selected in each group. Twenty-eight metabolites were found in common, including those from our multiomics signature, namely, tryptophan, glucose-6-phosphate, and P-Cresol-sulfate (Figure 7). Regarding PDR, the 2-methoxyresorcinol (fold change −1.33, VIP = 1.75) metabolite had the highest VIP, though it remained borderline with respect to significance in the *t*-test (*p* = 0.068). In the MO group, 10-Hydroxydecanoate (fold change = −1.28, VIP = 2.05, *p* = 0.009) and carnosine (fold change = −1.09, VIP = 1.78, *p* = 0.042) were found to be significantly downregulated compared to the control group (Table 3).

## 3. Discussion

Our aim was to identify metabolite markers that are complementary to known risk factors, such as glycemic control to improve existing risk stratification in DR-free patients with diabetes and those with the early stages of DR. Through a comprehensive untargeted multiomic approach coupling lipidomics and metabolomics profiling, we provide new insights in plasma metabolites and lipid species that differentiate patients with and without DR. In particular, phosphatidylcholines (PCs) were found to be dysregulated in the DR group, with an increase in alkyl-PCs (PC O-42:5) and a decrease in non-ether PCs (PC 14:0–16:1; PC 18:2–14:0). Interestingly, the metabolism of branched-chain amino acids (BCAAs) (methylglutaryl carnitine), the kynurenine pathway (tryptophan), and the and microbiota metabolism (p-Cresol sulfate) were found to have the ability to discriminate between patients with early stages of DR versus those with no or mild DR.

A wide selection of biofluids is already in use for multiomics analysis in human studies, including circulating blood (serum and plasma), eye fluids, and other samples [15]. Due to its easier availability and lower invasiveness, circulating blood is the most commonly used sample. It has the benefit of providing a global metabolomic signature which can be used in 3P (preventive, personalized, precision) medicine. In addition, plasma appear to have better reproducibility for detection of metabolite than serum [16]. As vitreous humor can directly reflect intraocular metabolic variations, tears can reflect the conditions of the oculi posterior segment; moreover, stool samples can reflect alterations in the fecal metabolome though the gut–retina axis [15]. However, the vitreous humor is a highly aqueous eye fluid interfacing with the retina, and can only be obtained from subjects with PDR during surgeries such as vitrectomy. Consequently, the volumes available for analysis are often small, making it difficult to establish a control group. In addition, vitreous hemorrhage can produce a massive influx of plasma metabolites into the vitreous fluid and can modify transcriptional activity in the retina. Barba et al. have evidenced the metabolic fingerprints (increase in glucose and lactate and decrease in galactitol and ascorbic acid) of the vitreous humor of PDR in patients living with type 1 diabetes (T1D), for which they used a ^1^H-NMR metabonomic approach [17]. The authors used non-diabetic patients with macular hole as a control group [17].

Biomarkers can offer an integrated understanding of a disease, such as from the pre-clinical to the most advanced stages. Excellent biomarkers should be specific and sensitive, be easily quantified in easy to take biological samples, and exhibit good linearity with the development of disease. Considering the complexity of the pathogenesis of DR, multiple biomarkers are seemingly more suitable than a single optimal one [18]. Regarding the metabolomics analysis, nuclear magnetic resonance (NMR) spectroscopy and mass spectrometry (MS) have both been developed. A significant advantage of NMR is the small number of samples required [19]. MS is often used in tandem with liquid chromatography (LC) or gas chromatography (GC), which are techniques applied to separate metabolites, thereby improving the resolution of isobaric compounds. In addition, LC-MS has become widely used in recent years, as MS has better sensitivity than NMR, allowing a wider spectrum of metabolites to be measured [18].

To date, a substantial amount of research has reported potential novel biomarkers for DR in human plasma in samples from patients with T1D or T2D with different disease durations and DR stages, including citrulline, indoleacetic acid, 1-methylhistidine, chenodeoxycholic acid, eicosapentaenoic acid, glutamine and glutamic acid, 1,5-gluconolactone, 2-deoxyribonic acid, gluconic acid, fumaric acid, uridine, acetic acid, and cytidine [13,18,20,21,22,23,24,25,26,27,28,29]. Several of these, such as 3,4-dihydroxybutyric acid (3,4-DHBA), were validated as independent risk markers for DR progression in a study of 648 individuals with T1D [27]. Most studies have included patients of South Asian ethnicity, and very few have adjusted their findings for potential confounders such as age, duration of diabetes, HbA1c, hypertension, diabetic kidney disease (DKD), and dyslipidemia.

Recently 12-hydroxyeicosatetraenoic acid (12-HETE) and 2-piperidone in serum exhibited better diagnostic performance than hemoglobin A1c (HbA1c) for differentiating DR from no DR in T2D, and showed high sensitivity towards early-stage DR [30]. Likewise, serum lipidomics and metabolomics profiling of hard exudates from 167 Chinese early-stage DR patients identified a lipid cluster enriched in triglycerides (29%), ceramides (17%), and N-acylethanolamines (15%) along with nineteen metabolites and thirteen pathways (taurine and hypotaurine metabolism, cysteine and methionine metabolism) [31]. In the present study, we have reported for the first time four new lipid clusters in Caucasian DR patients; we uniquely observed a specific upregulation of ether phosphatidylcholine, and in particular PC(O-42:5), in DR patients compared to controls. Clinical studies have provided limited and sometimes conflicting evidence on the relationships between circulating lipid levels and the development and progression of DR in people living with diabetes [32]. Moreover, certain lipid-lowering therapies implicating fibrates have shown protection against DR, although the effect was independent of changes in traditional blood lipid classes [33,34]. The retina is a highly specialised organ in which lipid levels are tightly regulated independently of their systemic levels. Lipid dysregulation can contribute to low-grade chronic inflammation and VEGF receptor 2 (VEGFR2) activation, resulting in increased retinal endothelial permeability and cell injury [35].

Purine metabolism, pyrimidine metabolism, arginine and proline metabolism, and glutamate metabolism are the most frequently reported differential pathways in DR metabolomics studies, with purine metabolism at the top (reported four times) in plasma samples [15,36]. We observed an increase in methylglutarylcarnitine, a key molecule in oxidation and metabolism of fatty acids, in the plasma of DR patients. Carnitine is essential for the transport of long-chain fatty acids into mitochondria via acylcarnitine intermediates prior to beta-oxidation. Our findings are consistent with previous studies [37,38].

Our study found increased levels of N-methyl-glutamate in the plasma of patients with DR. Previous studies have reported that the glutamine-to-glutamate ratio is the best distinctive metabolite for the presence of DR [13,23]. Glutamate is a key signal in the incretin-induced insulin secretion pathway, and is the major excitatory neurotransmitter in the central nervous system and retina [39,40]. It is necessary for the synthesis of key molecules such as glutathione as well as for polyglutamated folate cofactors, and plays a major role in signaling [40]. In the retina, glutamate is required for the transmission of visual signals from the photoreceptors to the ganglion cells. An increased level of glutamate in the retina may induce neurotoxic effects through the activation of its ionotropic receptors, as was found in a study of the rat retina, leading to uncontrolled intracellular calcium influx and cellular damage [41,42,43]. Meanwhile, in our study we observed that branched-chain amino acid (BCAAs) metabolism (valine, leucine, and isoleucine) was a remarkably KEGG-enriched pathway in DR. This is consistent with previous observations [36,44]. This could be linked to the neurotoxic effects of glutamate, as increased levels of BCAAs have been demonstrated to increase glutamate excitotoxicity by transamination of citric acid cycle intermediates [45]. Indeed, gabapentin, a leucine analogue and an inhibitor of branched-chain amino transferase (BCATc), has been shown to lower the retinal level of BCAAs, stimulate glutamate disposal, and ameliorate apoptosis and oxidative stress in diabetic rat retinas [45]. Therefore, more attention to this abnormal glutamate metabolism and BCAA metabolism is warranted in order to better understand the pathogenesis of DR.

In addition, we found that the plasma level of tryptophan was decreased in DR patients compared with controls. Ding et al. previously showed a decrease in tryptophan levels in 27 patients with PDR compared to 18 patients with non-proliferative DR. However, T2D disease duration was significantly higher in the PDR group, which could have impacted the results. In vitro studies using confluent cultures of a human retinal pigmented epithelial cell line (ARPE-19) have provided evidence that inducers of endoplasmic reticulum stress or nutrient deprivation in amino acids such as tryptophan or glutamine can directly lead to a marked increase in VEGF expression, suggesting that amino acid metabolism is critical in the response to hypoxia [46].

As DR is often associated with other microvascular diabetic complications, such as diabetic kidney disease (DKD) or macular oedema, we performed a subgroup analysis and identified specific plasma metabolomic signature of these conditions, with P-octopamine, pantothenate, deoxyguanosine-monophophate, and methylglutarylcarnitine being specific to DR only, N-methyl-2-pyridone-5-carboxamide, D-erythrose, DL-3-indolelactic acid, and adipate specific to DKD only, and methylguanidine, N-acetylneuraminate, arabinose, and mevalolactone specific to patients with both complications. Few metabolomic studies have accounted for DKD [15,36]. Tomofuji et al. revealed the serum metabolite signatures of patients with T2D and with both DR and DKD through a comprehensive nontargeted metabolomics approach combining capillary electrophoresis time-of-flight mass spectrometry (CE-TOFMS) and liquid chromatography TOFMS (LC-TOFMS). They compared the abundance of 364 serum metabolites between patients with T2D and with both DR and DKD (N = 141) and those without one of DR or DKD (N = 159). Interestingly, they evidenced N-acetylneuraminic acid, which is a major form of sialic acid in humans, as a relevant biomarker in T2D patients with both retinal and renal complications [47], which is consistent with our results.

Furthermore, we were able to evidence 10-hydroxydecanoate, carnosine, and methylguanidine as being specific to patients with macular oedema (MO), which is the most direct and important cause of visual impairment or blindness in people with DM. MO can occur at any stage of DR, and the severity of DR does not exactly match the severity of MO. Recently, Rhee et al. revealed that five plasma amino acids (asparagine, aspartic acid, glutamic acid, cysteine, and lysine), two organic compounds (citric acid and uric acid), and four oxylipins (12-oxoETE, 15-oxo-ETE, 9-oxoODE, and 20-carboxy leukotriene B4) can function as indicators for establishing a means of long-term prognosis associated with DME in long-standing T2DM patients (>15 years) of Korean origin [48].

These findings, together with aforementioned trends in amino acid levels, suggest that the plasma metabotype of DR is unique, and not a mere extension of the plasma metabotype of diabetic patients with microvascular complication. Furthermore, while not being exhaustive, these findings highlight once more some of the tissue specificity of the impact of diabetes and emphasize the need for global analysis to assess the tissue- or region-specific mechanisms at play in order to develop targeted interventions.

## 4. Limits

The large cohort of DR patients and diabetic controls were enrolled from the same institution with standardized sample collection and processing, which are strengths of the present study. However, most patients were Caucasian, meaning that the generalizability of these results has to be confirmed and the utility of the identified biomarkers has to be certified through cross-validation in different populations and ethnic groups. As cross-sectional sampling only captures a snapshot of plasma metabotypes, some of the identified markers may represent short-term metabolic perturbations instead of chronic risk factors associated with the development of DR. Moreover, the sensitivity and specificity of the diagnostic model should be validated in a prospective cohort. This study was a preliminary one and took a very discovery-oriented approach as part of the multicentric Angiosafe T2D study (NCT02671864), which aims to include 7200 patients, who will be followed up at three years to evaluate DR presence, incidence, and progression. At present, not all of the patients have been reevaluated three years after their inclusion. Hence, the confirmation study plan is to validate the potential biomarkers identified in this proof-of-concept study in the patients who have DR progression between inclusion and the three-year follow-up visit. Therefore, these findings provide the foundation for longitudinal metabonomic studies to establish the correlation and predictive value of metabolite and lipid profiles identified with the rate of DR progression in patients with T2D. Future studies should consider using other biological matrices (e.g., urine, cerebrospinal fluid) to expand and confirm these findings.

## 5. Materials and Methods

A total of 200 T2D subjects, including 100 who had DR (moderate, severe, or proliferative, with or without macular oedema) and 100 patients without DR or with mild DR, were randomly selected from among the Angiosafe T2D cohort and matched in terms of age (±5 years), sex, duration of diabetes (±2 years), HbA1c (±0.5%), and hypertension. The Angiosafe T2D cohort (NCT02671864) is extensively described elsewhere [8] and aims to include 7200 patients who will be followed up at 3 years to evaluate DR presence, incidence, or progression according to the international classification of Diabetic Retinopathy [49]. For this study, we included and analyzed patients were enrolled at the Endocrinology, Metabolic Diseases, and Nutrition Department, Pole ENDO, APHM, Marseille.

### 5.1. Retinal Imaging

Photographs (45°, 2-field or 9-field) were taken of both eyes by a trained nurse using a CANON CR-2 fundus camera [8]. Two grading teams of independent observers, each consisting of a primary recently qualified ophthalmologist and a second more experienced ophthalmologist, were assigned to each patient. Graders assessed all photographs for DR according to the international classification of Diabetic Retinopathy [49]. Macular oedema was diagnosed on optical coherence tomography as retinal thickening of one or more disc area/s (with any part lying less than a disc diameter from the fovea) or hard exudates less than 500 μ from the fovea.

### 5.2. Sample Collection

Blood samples were collected using EDTA collection tubes and the plasma was separated and stored at −80 °C until extraction. Plasma was extracted using two separate extraction protocols to perform untargeted metabolomics and lipidomics analyses.

### 5.3. Solvents

Ultrapure water was obtained from a Milli-Q system (Millipore, MA, USA). Methanol, isopropanol, chloroform, methyl-terbutyl ether, and acetonitrile were of LC–MS or HPLC grade (Carlo Erba Reagents, Val de Reuil, France).

### 5.4. Extraction

For metabolomics analysis, plasma samples (50 µL) were homogenized with 200 µL of cold methanol (−20 °C), thoroughly shaken for 60 s, and incubated for 30 min at −20 °C to precipitate proteins. Samples were then centrifuged at 11,000 rpm and 4 °C for 15 min and the supernatants were collected and centrifuged through a 10 kDa microcentrifuge filter (VWR, Rosny sous Bois, France) for 45 min under the same conditions, dried under nitrogen flow, and stored at −80 °C until analysis. Dried extracts were dissolved in 125 µL water:acetonitrile (1:1 *v*/*v*) For lipidomics analysis, tertbutyl-methyl ether was used as the extraction solvent [50]. Briefly, 750 µL of methanol and 2.5 mL of tertbutyl-methyl ether were added to 100 µL of plasma and vortexed for 1 h, then 625 µL of deionized water was added to each sample and the tubes were incubated for 10 min after homogenization at room temperature. The tubes were centrifuged for 10 min at 1000 rpm 10 °C and the upper organic phase was placed in another tube; 2 mL of a tertbutyl-methyl ether/methanol/water mixture (20:6:5, *v*/*v*/*v*) was added to the lower phase and centrifuged as previously described for a second extraction. The upper organic phase was pooled with the one obtained in the first extraction. Samples were evaporated under a stream of nitrogen and the dried lipid extracts were stored at −80 °C until processing. Lipid extracts were then resuspended in 200 µL of mobile phase mixture (A: 65%, D: 35%, *v*/*v*). For both analyses, 25 μL of each sample was combined to obtain quality control (QC) samples.

Samples were randomly assigned to the injection table and interspaced (1 of 5) with QC samples or solvent for blank. All samples were analyzed in a single series for metabolomics or lipidomics analysis.

### 5.5. Metabolomics LC-MS

LC-MS analyses were performed with a Dionex Ultimate 3000 (Thermo Scientific, Courtaboeuf, France) ultra-performance liquid chromatography (UPLC) system coupled with a Thermo Q Exactive Plus mass spectrometer (MS). A reverse-phase (RP) Hypersil Gold (100 mm × 2.1 mm × 1.9 µm) (Thermo Scientific, France) column and a hydrophilic SeQuant^®^ ZIC-HILIC Peek Coated (150 × 2.1 mm × 5 µm) (Merck Millipore, MA, USA) column were used for compound separation to ensure broad coverage of the metabolome. The detailed LC/MS conditions are described in [51].

### 5.6. Lipidomics LC-MS

UHPLC separation was performed on a Vanquish Horizon device (Thermo Fisher Scientific, Courtaboeuf, France) using an Accucore C18 column (150 × 2.1 mm, 2.6 μm). The column temperature was kept at 45 °C. Mobile phase A contained 10 mmol/L ammonium formate in 60% acetonitrile and 0.1% formic acid, while mobile phase D contained a 10 mmol/L ammonium formate in acetonitrile:propan-2-ol (1:9, *v*/*v*) mixture with 0.1% formic acid. The flow rate was 0.4 mL/min. The elution gradient was as follows: 35% D at the beginning, 35% to 60% D for 4 min, 60% to 85% B for 8 min, 85% to 100% B for 9 min, 100% B for 3 min, and 35% B for 4 min. The injection volume was 4 μL for both ionization modes. Samples were randomly assigned to the injection table and interspaced (1 of 5) with quality control samples made up of a pool of each sample or solvent for blank.

Full scan mass spectra were acquired using an Orbitrap Exploris 240 (Thermo Fisher) mass spectrometer in positive and negative ionization modes, acquiring data within the 150 to 1500 *m*/*z* range. Briefly, the drying temperature was set to 200 °C and the ion transfer tube temperature to 320 °C. The capillary voltage was set to 3000 V for the positive ionization mode and 3100 V for the negative ionization mode, while the RF lens was set to 70%. The sheath, auxiliary, and sweep gases were set tot 60, 20, and 1 L/min, respectively. The resolving power of the orbitrap mass analyzer was set to 120,000 FWHM for *m*/*z* 200 with a 3 Hz scan rate, and the maximum injection time was set to 200 ms. Full scan mass spectra were acquired for each sample.

MS/MS spectra were acquired using the AcquireX Intelligent Data Acquisition Workflow based on data-dependent analysis for the acquisition of MS/MS spectra. This workflow was applied on a pooled sample, one control and one randomly selected DR paired sample. The Deep Scan AcquireX workflow creates an exclusion list of ions presents in a blank sample and an inclusion list of ions presents in a sample of interest, then exhaustively fragments all ions from the inclusion list together with ions detected under the applied conditions in four consecutive sample injections. Ions fragmented in the first MS/MS analysis are systematically added to the exclusion list and non-fragmented low abundance ions are fragmented. This allows for exhaustive fragmentation and identification of the lipidome. Full scan and DDA MS/MS spectra were acquired using a resolving power of 60,000 FWHM and 15,000 FWHM, respectively. Ions under 10,000 absolute intensity were considered as noise. Ions with higher intensity were fragmented in decreasing order of intensity in a 3 s cycle, with up to 50 ms used for the fragmentation of one ion.

### 5.7. Data Treatment

Raw LC–MS data were converted to the mzXML file format, and peak detection and alignment were performed using the XCMS script in R (R version 3.6.3). Four main steps were applied: peak picking, peak grouping (alignment), retention time correction, and a second peak grouping step. The centWave method was used to extract peaks and a nonlinear LOESS normalization method [52] was used to correct the analytical drift with Workflow4Metabolomics [53]. After normalization, further filtering was performed by calculating the coefficient of variation of variable intensity in the QC samples, with the cutoff set at <30%.

### 5.8. Annotations

Feature annotation for metabolomics analysis (HILIC and RP) was performed using in-house libraries of approximately 800 standards under the same conditions [51], while lipid annotation was performed by MS/MS spectral matching using LipidSearch software (Thermo Scientific, France). Each annotated metabolite was assigned a biological role based on the Human Metabolome Database (www.hmdb.ca), PubChem description, and KEGG pathways. Complementary information in PubMed publications was used whenever available. The annotated metabolites reported in the Supplementary Data were then grouped according to their functional role.

### 5.9. Statistical Analyses

Anthropometric and biochemical parameters were expressed as mean ± SD or median and as 25th to 75th percentile if not parametric. The normal distribution of datasets was assessed using the Shapiro–Wilk normality test. Significant differences between groups were determined using the paired Student’s *t*-test or Mann–Whitney test where appropriate. The Chi-2 McNemar test was used to compare categorical variables between groups. Statistical analyses were performed with Prism 9 (Graphpad, MA, USA).

For metabolomic data, features from both ionization modes for the HILIC and RP columns were combined into a single dataset, while for lipidomic data both ionization modes were combined and analyzed separately. Principal component analysis (PCA) was performed after each preprocessing step to view the data and detect outliers. All data were normalized using Probability Quotient Normalization prior to statistical analysis. PCA, partial least square discriminant analyses (PLS-DA), the variable of importance in Projection (VIP) method, and hierarchical PLS-DA were performed using SIMCA 17 software (Sartorius, Aubagne, France). Models were validated by cross-validation using ANOVA and a permutation procedure to check for overfitting through permutation tests (200 permutations). Univariate statistical analysis, hierarchical clustering, heatmapping, and pathway enrichment were performed using the online tool MetaboAnalyst 5.0 [54], while partial correlations were calculated with the R package GeneNet and network visualization was performed using Cytoscape.

Hierarchical PLS-DA was performed based on the contributions of separate orthogonal PLS-DAs calculated from every functional set of metabolites, allowing a composite score value to be generated for each functional dataset [55]. Multiblock PLS and hierarchical PLS enabled aggregation of the data into metabolic function blocks to ease data interpretation and biological understanding of the role of DR. The functional metabolic blocks were weighted to consider the number of metabolites per block [56]. For lipid blocking, lipid species were grouped according to clusters calculated by hierarchical clustering analysis (Ward method). The score values of lipid blocks were generated by hierarchical-PLS-DA as described previously [57]. Scores from the hierarchical PLS-DA multiblock analysis were analysed to determine the most significant biological functions related to the clinical outcome. For metabolomics and lipidomics analyses, the Benjamini–Yekutieli procedure for controlling the false discovery rate was applied. *p* < 0.05 was considered statistically significant.

## 6. Conclusions

In conclusion, significant variation in plasma metabolites and lipids was found in T2D patients with DR compared to T2D patients without DR. Panels of molecules identified by metabolomics and lipidomics profiling could potentially be relevant biomarkers for the diagnosis of DR, although there is a need for validation studies in order to confirm their role in identifying patients at risk of DR progression. Further investigation is required in order to quantitatively detect candidate metabolites in an expanded cohort. Nevertheless, our work demonstrates that this multiomic approach should in the near future enable monitoring of the appearance of disease and disease progression at an early stage. This could help clinicians and ophthalmologists to adapt the frequency of retinal screening in this period of lack of human resources for eye health. The most prominent advances in diagnosis and treatment have been made for later stages of the disease. Robust and specific risk markers for onset and early progression of DR remain lacking, however, and need further research. The identification of specific signatures associated with PDR, macular oedema, and DR associated with chronic kidney disease can provide valuable insights into the pathophysiology of these conditions, and could potentially guide the development of targeted therapies. Future studies should consider ways to confirm these findings in other biological matrices as well.

## Figures and Tables

**Table 1:** Clinical and biological characteristics of the study population.

	Control (*n* = 97)	DR (*n* = 97)	*p* Value
Age (years)	64.3 ± 8.7	64.4 ± 8.9	0.778
Sex (M/F, %M)	66/31 (68)	66/31 (68)	>0.999
Body mass index (kg/m^2^)	29.8 [26.6;32.8]	29.1 [26.3;34.1]	0.553
Diabetes duration (years)	16.9 ± 9.3	16.9 ± 9.4	>0.999
Hypertension (*n*, %)	80 (82)	80 (82)	>0.999
Dyslipidemia (*n*, %)	67 (69)	68 (70)	>0.999
Nephropathy (*n*, %)	36 (37)	53 (55)	0.021
GFR (mL/min/1.73 m^2^)	90 [73;103]	82 [62;97]	<0.001
HbA1c (%)	7.8 [7.0;8.5]	7.8 [7.0;8.6]	0.824
Cholesterol (g/L)	1.61 [1.37;1.86]	1.47 [1.23;1.76]	0.051
HDL-Chol (g/L)	0.40 [0.34;0.48]	0.43 [0.36;0.53]	0.279
LDL-Chol (g/L)	0.87 [0.63;1.09]	0.74 [0.57;1.01]	0.172
Triglycerides (g/L)	1.40 [1.02;2.04]	1.29 [0.79;1.77]	0.099
ASAT (UI/L)	21 [18;28]	23 [18;29]	0.230
ALAT (UI/L)	24 [16;35]	23 [17;31]	0.240
GGT (UI/L)	29 [21;43]	32 [21;64]	0.109

Nephropathy: Urine albumin/creatinine ratio ≥ 30 mg/g or urine protein/creatinine ratio ≥ 300 mg/g or GFR ≤ 60 mL/min/1.73 m^2^; GFR: glomerular filtration rate; Chol: Cholesterol.

**Table 2:** Top four metabolites implicated in diabetic complications.

Specific to Diabetic Retinopathy Only (DR)			
Metabolites	Fold Change	VIP	*p* Value
P-octopamine	−1.18	2.83	0.014
Pantothenate	1.25	2.51	0.034
Deoxyguanosine-monophophate	1.48	2.37	0.019
Methylglutarylcarnitine	1.86	2.12	0.037
**Specific to Diabetic Kidney Disease Only (DKD)**			
**Metabolites**	**Fold Change**	**VIP**	***p* Value**
N-methyl-2-pyridone-5-carboxamide	1.52	2.59	<0.001
D-Erythrose	1.21	2.26	0.002
DL-3-Indolelactic Acid	1.26	2.17	<0.001
Adipate	−1.18	2.09	0.007
**Specific to Diabetic Retinopathy Associated to Diabetic Kidney Disease (DR + DKD)**			
**Metabolites**	**Fold Change**	**VIP**	***p* Value**
Methylguanidine	1.89	2.42	<0.001
N-acetylneuraminate	1.73	2.37	<0.001
Arabinose	1.70	2.37	<0.001
Mevalolactone	1.30	2.32	<0.001

**Table 3:** Top three VIP metabolites in diabetic eye disease.

Specific to Proliferative Diabetic Retinopathy			
Metabolites	Fold Change	VIP	*p* Value
2-Methoxyresorcinol	−1.28	1.75	ns
α-Hydroxyisobutyrate	1.07	1.75	ns
2-Hydroxycaproic acid	1.10	1.60	ns
**Specific to Macular Oedema**			
**Metabolites**	**Fold Change**	**VIP**	***p* Value**
10-Hydroxydecanoate		2.05	0.009
Carnosine		1.78	0.042
Methylguanidine		1.63	ns

## Data Availability

The data presented in this study are available on reasonable request from the corresponding author.

## Supplementary Materials

The following supporting information can be downloaded at: https://www.mdpi.com/article/10.3390/ijms241512053/s1.

## References

[1] Teo Z.L., Tham Y.-C., Yu M., Chee M.L., Rim T.H., Cheung N., Bikbov M.M., Wang Y.X., Tang Y., Lu Y. (2021). Global Prevalence of Diabetic Retinopathy and Projection of Burden through 2045: Systematic Review and Meta-Analysis. Ophthalmology.

[2] Yau J.W.Y., Rogers S.L., Kawasaki R., Lamoureux E.L., Kowalski J.W., Bek T., Chen S.-J., Dekker J.M., Fletcher A., Grauslund J. (2012). Global Prevalence and Major Risk Factors of Diabetic Retinopathy. Diabetes Care.

[3] Perais J., Agarwal R., Evans J.R., Loveman E., Colquitt J.L., Owens D., Hogg R.E., Lawrenson J.G., Takwoingi Y., Lois N. (2023). Prognostic Factors for the Development and Progression of Proliferative Diabetic Retinopathy in People with Diabetic Retinopathy. Cochrane Database Syst. Rev..

[4] Hirsch I.B., Brownlee M. (2010). Beyond Hemoglobin A1c--Need for Additional Markers of Risk for Diabetic Microvascular Complications. JAMA.

[5] Kirthi V., Nderitu P., Alam U., Evans J.R., Nevitt S., Malik R.A., Hopkins D., Jackson T.L. (2022). The Prevalence of Retinopathy in Prediabetes: A Systematic Review. Surv. Ophthalmol..

[6] Kiziltoprak H., Tekin K., Inanc M., Goker Y.S. (2019). Cataract in Diabetes Mellitus. World J. Diabetes.

[7] Zikhali T., Kalinda C., Xulu-Kasaba Z.N. (2022). Screening of Diabetic Retinopathy Using Teleophthalmology to Complement Human Resources for Eye Health: A Systematic Review and Meta-Analysis. Clin. Pract..

[8] Gaborit B., Julla J.-B., Besbes S., Proust M., Vincentelli C., Alos B., Ancel P., Alzaid F., Garcia R., Mailly P. (2020). Glucagon-like Peptide 1 Receptor Agonists, Diabetic Retinopathy and Angiogenesis: The AngioSafe Type 2 Diabetes Study. J. Clin. Endocrinol. Metab..

[9] Newgard C.B. (2017). Metabolomics and Metabolic Diseases: Where Do We Stand?. Cell Metab..

[10] Jeppesen M.J., Powers R. (2023). Multiplatform Untargeted Metabolomics. Magn. Reson. Chem..

[11] Chu S.H., Huang M., Kelly R.S., Benedetti E., Siddiqui J.K., Zeleznik O.A., Pereira A., Herrington D., Wheelock C.E., Krumsiek J. (2019). Integration of Metabolomic and Other Omics Data in Population-Based Study Designs: An Epidemiological Perspective. Metabolites.

[12] Yang K., Han X. (2016). Lipidomics: Techniques, Applications, and Outcomes Related to Biomedical Sciences. Trends Biochem. Sci..

[13] Ding C., Wang N., Wang Z., Yue W., Li B., Zeng J., Yoshida S., Yang Y., Zhou Y. (2022). Integrated Analysis of Metabolomics and Lipidomics in Plasma of T2DM Patients with Diabetic Retinopathy. Pharmaceutics.

[14] Oltvai Z.N., Barabási A.-L. (2002). Systems Biology. Life’s Complexity Pyramid. Science.

[15] Jian Q., Wu Y., Zhang F. (2022). Metabolomics in Diabetic Retinopathy: From Potential Biomarkers to Molecular Basis of Oxidative Stress. Cells.

[16] Yu Z., Kastenmüller G., He Y., Belcredi P., Möller G., Prehn C., Mendes J., Wahl S., Roemisch-Margl W., Ceglarek U. (2011). Differences between Human Plasma and Serum Metabolite Profiles. PLoS ONE.

[17] Barba I., Garcia-Ramírez M., Hernández C., Alonso M.A., Masmiquel L., García-Dorado D., Simó R. (2010). Metabolic Fingerprints of Proliferative Diabetic Retinopathy: An 1H-NMR-Based Metabonomic Approach Using Vitreous Humor. Investig. Ophthalmol. Vis. Sci..

[18] Du X., Yang L., Kong L., Sun Y., Shen K., Cai Y., Sun H., Zhang B., Guo S., Zhang A. (2022). Metabolomics of Various Samples Advancing Biomarker Discovery and Pathogenesis Elucidation for Diabetic Retinopathy. Front. Endocrinol..

[19] Laíns I., Gantner M., Murinello S., Lasky-Su J.A., Miller J.W., Friedlander M., Husain D. (2019). Metabolomics in the Study of Retinal Health and Disease. Prog. Retin. Eye Res..

[20] Xia J.-F., Wang Z., Liang Q.-L., Wang Y.-M., Li P., Luo G.-A. (2011). Correlations of Six Related Pyrimidine Metabolites and Diabetic Retinopathy in Chinese Type 2 Diabetic Patients. Clin. Chim. Acta.

[21] Xia J., Wang Z., Zhang F. (2014). Association between Related Purine Metabolites and Diabetic Retinopathy in Type 2 Diabetic Patients. Int. J. Endocrinol..

[22] Chen L., Cheng C.-Y., Choi H., Ikram M.K., Sabanayagam C., Tan G.S.W., Tian D., Zhang L., Venkatesan G., Tai E.S. (2016). Plasma Metabonomic Profiling of Diabetic Retinopathy. Diabetes.

[23] Rhee S.Y., Jung E.S., Park H.M., Jeong S.J., Kim K., Chon S., Yu S.-Y., Woo J.-T., Lee C.H. (2018). Plasma Glutamine and Glutamic Acid Are Potential Biomarkers for Predicting Diabetic Retinopathy. Metabolomics.

[24] Zhu X.-R., Yang F.-Y., Lu J., Zhang H.-R., Sun R., Zhou J.-B., Yang J.-K. (2019). Plasma Metabolomic Profiling of Proliferative Diabetic Retinopathy. Nutr. Metab..

[25] Sun Y., Zou H., Li X., Xu S., Liu C. (2021). Plasma Metabolomics Reveals Metabolic Profiling for Diabetic Retinopathy and Disease Progression. Front. Endocrinol..

[26] Lin H.-T., Cheng M.-L., Lo C.-J., Lin G., Lin S.-F., Yeh J.-T., Ho H.-Y., Lin J.-R., Liu F.-C. (2019). 1H Nuclear Magnetic Resonance (NMR)-Based Cerebrospinal Fluid and Plasma Metabolomic Analysis in Type 2 Diabetic Patients and Risk Prediction for Diabetic Microangiopathy. J. Clin. Med..

[27] Curovic V.R., Suvitaival T., Mattila I., Ahonen L., Trošt K., Theilade S., Hansen T.W., Legido-Quigley C., Rossing P. (2020). Circulating Metabolites and Lipids Are Associated to Diabetic Retinopathy in Individuals with Type 1 Diabetes. Diabetes.

[28] Wang Z., Tang J., Jin E., Ren C., Li S., Zhang L., Zhong Y., Cao Y., Wang J., Zhou W. (2022). Metabolomic Comparison Followed by Cross-Validation of Enzyme-Linked Immunosorbent Assay to Reveal Potential Biomarkers of Diabetic Retinopathy in Chinese with Type 2 Diabetes. Front. Endocrinol..

[29] Peters K.S., Rivera E., Warden C., Harlow P.A., Mitchell S.L., Calcutt M.W., Samuels D.C., Brantley M.A. (2022). Plasma Arginine and Citrulline Are Elevated in Diabetic Retinopathy. Am. J. Ophthalmol..

[30] Xuan Q., Ouyang Y., Wang Y., Wu L., Li H., Luo Y., Zhao X., Feng D., Qin W., Hu C. (2020). Multiplatform Metabolomics Reveals Novel Serum Metabolite Biomarkers in Diabetic Retinopathy Subjects. Adv. Sci..

[31] Shen Y., Wang H., Fang J., Liu K., Xu X. (2023). Novel Insights into the Mechanisms of Hard Exudate in Diabetic Retinopathy: Findings of Serum Lipidomic and Metabolomics Profiling. Heliyon.

[32] Jenkins A.J., Grant M.B., Busik J.V. (2022). Lipids, Hyperreflective Crystalline Deposits and Diabetic Retinopathy: Potential Systemic and Retinal-Specific Effect of Lipid-Lowering Therapies. Diabetologia.

[33] Keech A.C., Mitchell P., Summanen P.A., O’Day J., Davis T.M.E., Moffitt M.S., Taskinen M.-R., Simes R.J., Tse D., Williamson E. (2007). Effect of Fenofibrate on the Need for Laser Treatment for Diabetic Retinopathy (FIELD Study): A Randomised Controlled Trial. Lancet.

[34] Chew E.Y., Ambrosius W.T., Davis M.D., Danis R.P., Gangaputra S., Greven C.M., Hubbard L., Esser B.A., ACCORD Study Group, ACCORD Eye Study Group (2010). Effects of Medical Therapies on Retinopathy Progression in Type 2 Diabetes. N. Engl. J. Med..

[35] Eid S., Sas K.M., Abcouwer S.F., Feldman E.L., Gardner T.W., Pennathur S., Fort P.E. (2019). New Insights into the Mechanisms of Diabetic Complications: Role of Lipids and Lipid Metabolism. Diabetologia.

[36] Arneth B., Arneth R., Shams M. (2019). Metabolomics of Type 1 and Type 2 Diabetes. Int. J. Mol. Sci..

[37] Sumarriva K., Uppal K., Ma C., Herren D.J., Wang Y., Chocron I.M., Warden C., Mitchell S.L., Burgess L.G., Goodale M.P. (2019). Arginine and Carnitine Metabolites Are Altered in Diabetic Retinopathy. Investig. Opthalmol. Vis. Sci..

[38] Paris L.P., Johnson C.H., Aguilar E., Usui Y., Cho K., Hoang L.T., Feitelberg D., Benton H.P., Westenskow P.D., Kurihara T. (2016). Global Metabolomics Reveals Metabolic Dysregulation in Ischemic Retinopathy. Metabolomics.

[39] Gheni G., Ogura M., Iwasaki M., Yokoi N., Minami K., Nakayama Y., Harada K., Hastoy B., Wu X., Takahashi H. (2014). Glutamate Acts as a Key Signal Linking Glucose Metabolism to Incretin/CAMP Action to Amplify Insulin Secretion. Cell Rep..

[40] Brosnan J.T., Brosnan M.E. (2013). Glutamate: A Truly Functional Amino Acid. Amino Acids.

[41] Bogdanov P., Corraliza L., Villena J.A., Carvalho A.R., Garcia-Arumí J., Ramos D., Ruberte J., Simó R., Hernández C. (2014). The Db/Db Mouse: A Useful Model for the Study of Diabetic Retinal Neurodegeneration. PLoS ONE.

[42] Ng Y.-K., Zeng X.-X., Ling E.-A. (2004). Expression of Glutamate Receptors and Calcium-Binding Proteins in the Retina of Streptozotocin-Induced Diabetic Rats. Brain Res..

[43] Santiago A.R., Gaspar J.M., Baptista F.I., Cristóvão A.J., Santos P.F., Kamphuis W., Ambrósio A.F. (2009). Diabetes Changes the Levels of Ionotropic Glutamate Receptors in the Rat Retina. Mol. Vis..

[44] Bloomgarden Z. (2018). Diabetes and Branched-Chain Amino Acids: What Is the Link?. J. Diabetes.

[45] Ola M.S., Alhomida A.S., LaNoue K.F. (2019). Gabapentin Attenuates Oxidative Stress and Apoptosis in the Diabetic Rat Retina. Neurotox. Res..

[46] Abcouwer S.F., Marjon P.L., Loper R.K., Vander Jagt D.L. (2002). Response of VEGF Expression to Amino Acid Deprivation and Inducers of Endoplasmic Reticulum Stress. Investig. Ophthalmol. Vis. Sci..

[47] Tomofuji Y., Suzuki K., Kishikawa T., Shojima N., Hosoe J., Inagaki K., Matsubayashi S., Ishihara H., Watada H., Ishigaki Y. (2023). Identification of Serum Metabolome Signatures Associated with Retinal and Renal Complications of Type 2 Diabetes. Commun. Med..

[48] Rhee S.Y., Jung E.S., Suh D.H., Jeong S.J., Kim K., Chon S., Yu S.-Y., Woo J.-T., Lee C.H. (2021). Plasma Amino Acids and Oxylipins as Potential Multi-Biomarkers for Predicting Diabetic Macular Edema. Sci. Rep..

[49] Wilkinson C.P., Ferris F.L., Klein R.E., Lee P.P., Agardh C.D., Davis M., Dills D., Kampik A., Pararajasegaram R., Verdaguer J.T. (2003). Proposed International Clinical Diabetic Retinopathy and Diabetic Macular Edema Disease Severity Scales. Ophthalmology.

[50] Matyash V., Liebisch G., Kurzchalia T.V., Shevchenko A., Schwudke D. (2008). Lipid Extraction by Methyl-Tert-Butyl Ether for High-Throughput Lipidomics. J. Lipid Res..

[51] Grison S., Habchi B., Gloaguen C., Kereselidze D., Elie C., Martin J.-C., Souidi M. (2022). Early Metabolomic Markers of Acute Low-Dose Exposure to Uranium in Rats. Metabolites.

[52] Van der Kloet F.M., Bobeldijk I., Verheij E.R., Jellema R.H. (2009). Analytical Error Reduction Using Single Point Calibration for Accurate and Precise Metabolomic Phenotyping. J. Proteome Res..

[53] Giacomoni F., Le Corguillé G., Monsoor M., Landi M., Pericard P., Pétéra M., Duperier C., Tremblay-Franco M., Martin J.-F., Jacob D. (2015). Workflow4Metabolomics: A Collaborative Research Infrastructure for Computational Metabolomics. Bioinformatics.

[54] Pang Z., Zhou G., Ewald J., Chang L., Hacariz O., Basu N., Xia J. (2022). Using MetaboAnalyst 5.0 for LC-HRMS Spectra Processing, Multi-Omics Integration and Covariate Adjustment of Global Metabolomics Data. Nat. Protoc..

[55] Martin J.-C., Berton A., Ginies C., Bott R., Scheercousse P., Saddi A., Gripois D., Landrier J.-F., Dalemans D., Alessi M.-C. (2015). Multilevel Systems Biology Modeling Characterized the Atheroprotective Efficiencies of Modified Dairy Fats in a Hamster Model. Am. J. Physiol. Heart Circ. Physiol..

[56] Wold S., Kettaneh N., Tjessem K. (1996). Hierarchical Multiblock PLS and PC Models for Easier Model Interpretation and as an Alternative to Variable Selection. J. Chemom..

[57] Fraser K., Roy N.C., Goumidi L., Verdu A., Suchon P., Leal-Valentim F., Trégouët D.-A., Morange P.-E., Martin J.-C. (2020). Plasma Biomarkers and Identification of Resilient Metabolic Disruptions in Patients with Venous Thromboembolism Using a Metabolic Systems Approach. Arterioscler. Thromb. Vasc. Biol..

